# Increased transmissibility of SARS-CoV-2 alpha variant (B.1.1.7) in children: three large primary school outbreaks revealed by whole genome sequencing in the Netherlands

**DOI:** 10.1186/s12879-022-07623-9

**Published:** 2022-08-29

**Authors:** Koen M. F. Gorgels, Lieke B. van Alphen, Brian M. J. W. van der Veer, Volker H. Hackert, Audrey Y. J. Hensels, Casper D. J. den Heijer, Jozef Dingemans, Paul H. M. Savelkoul, Christian J. P. A. Hoebe

**Affiliations:** 1 Department of Sexual Health, Infectious Diseases and Environmental Health, South Limburg Public Health Service, PO Box 33, 6400 AA Heerlen, The Netherlands; 2 Department of Medical Microbiology, Care and Public Health Research Institute (CAPHRI), Faculty of Health, Medicine and Life Sciences, Maastricht University Medical Centre (MUMC+), P.O. Box 5800, 6202 AZ Maastricht, The Netherlands; 3 Department of Social Medicine, Care and Public Health Research Institute (CAPHRI), Faculty of Health, Medicine and Life Sciences, Maastricht University, P.O. Box 616, 6200 MD Maastricht, The Netherlands

**Keywords:** SARS-CoV-2, Outbreak, Primary school, B.1.1.7 variant, Infection prevention and control, Transmissibility in children

## Abstract

**Background:**

Variant of concern (VOC) SARS-CoV-2 alpha variant (B.1.1.7) was the dominant strain in the Netherlands between March 2021–June 2021. We describe three primary school outbreaks due to the alpha variant using whole genome sequencing with evidence of large-scale transmission among children, teachers and their household contacts.

**Method:**

All outbreaks described were investigated by the South Limburg Public Health Service, the Netherlands. A case was defined as an individual with a real-time polymerase chain reaction test or antigen test positive for SARS-CoV-2. Whole genome sequencing was performed on random samples from at least one child and one teacher of each affected class.

**Results:**

Peak attack rates in classes were 53%, 33% and 39%, respectively. Specific genotypes were identified for each school across a majority of affected classes. Attack rates were high among staff members, likely to promote staff-to-children transmission. Cases in some classes were limited to children, indicating child-to-child transmission. At 39%, the secondary attack rate (SAR) in household contacts of infected children was remarkably high, similar to SAR in household contacts of staff members (42%). SAR of household contacts of asymptomatic children was only 9%.

**Conclusion:**

Our findings suggest increased transmissibility of the alpha variant in children compared to preceding non-VOC variants, consistent with a substantial rise in the incidence of cases observed in primary schools and children aged 5–12 since the alpha variant became dominant in March 2021. Lack of mandatory masking, insufficient ventilation and lack of physical distancing also probably contributed to the school outbreaks. The rise of the delta variant (B.1.617.2) since July 2021 which is estimated to be 55% more transmissible than the alpha variant, provides additional urgency to adequate infection prevention in school settings.

**Supplementary Information:**

The online version contains supplementary material available at 10.1186/s12879-022-07623-9.

## Background

Variant of concern (VOC) SARS-CoV-2 alpha variant (B.1.1.7) was the dominant strain in the Netherlands between March 2021 – June 2021. This variant has been associated with increased transmissibility, but evidence in children of school age is limited [1]. Preceding the alpha variant, reports of SARS-CoV-2 transmission in schools were uncommon and in-school transmission appeared to be low although one study found many transmission events occurring in school [2, 3]. A German study on outbreaks with the alpha variant in three day care centers for children suggests increased susceptibility and infectiousness in children aged 0–4 years, but no outbreaks in primary schools with the alpha variant have been reported [4].

In the Netherlands primary schools were closed from December 17 and reopened on February 9. All children with COVID-19 related symptoms were advised to stay at home and undergo testing. No masking was recommended but schools were free to mandate masks in staff members and older children in grade 7–8. Keeping 1.5 m distance was recommended between staff members but not between children and extracurricular indoor sports were prohibited for children of all ages. Despite these measures multiple large outbreaks occurred. Here, we describe three large outbreaks caused by three different genotypes of the alpha variant. We provide a detailed description of the infection prevention measures that were put in place and the secondary attack rates (SARs) in household contacts. We also present these outbreaks towards the background of primary school cases, circulating variants and overall community incidence.

## Methods

### Epidemiological investigation

Using a retrospective observational study, we investigated three primary school outbreaks (school 1, 2 and 3) in March–April 2021, following the reopening of primary schools on February 9. The total numbers of children ranged between 265–529 distributed over 9–19 classes, with 19–34 children per class. The schools housed children from grade 1–8 with ages ranging between 4 and 12 years old. Infection prevention at the time of the outbreaks included the use of medical face masks by teachers outside the classroom, availability of disinfectant hand gel in every classroom used prior to entering the classroom, and supervised cohorting of each individual class during lunch breaks with children (school 1 and 2 only). Inside the classroom no masks were used by staff members. Students did not wear masks inside or outside the classroom. No physical distance was maintained between students of the same class. No online schooling was given during the study period. All staff and/or parent meetings were held online. All outbreaks described were investigated by the South Limburg Public Health Service, the Netherlands. Additional information on infection prevention measures at the schools was obtained through visits at affected schools and interviews with concerned managerial staff.

### Case definition of cases at schools

A case was defined as an individual with a real-time polymerase chain reaction (RT-PCR) test or antigen test positive for SARS-CoV-2. Cases were defined as symptomatic if they reported COVID-19 related symptoms, including common cold symptoms (nasal cold, runny nose, sneezing or sore throat), cough, elevated temperature or fever (temperature > 38 °C), loss of taste or smell, diarrhea, nausea, fatigue and headache [5]. Cases were defined as asymptomatic if they reported none of these symptoms at the time of their positive test and developed no symptoms in the seven days that followed.

The contagious period in symptomatic cases was defined as the two days preceding symptom development. In asymptomatic and pre-symptomatic cases, the date of the positive test was used as a proxy for disease onset. Cases were further classified into two different categories, i.e., children and staff members. Staff members were defined as teachers and managerial staff of affected schools. An outbreak was considered finished when no new cases were reported over a period of 28 days (two times maximum incubation period of SARS-Cov-2). Attack rate (AR) was calculated as the number children cases divided by the total number of children each class. In each class we assessed if a teacher had taught a class with positively tested children.

### Case definition of secondary cases at households

Household members of cases were asked to quarantine at home and were advised to test when developing symptoms and on day 5 following last contact with the case. All household contacts testing positive within 14 days after diagnosis of the primary case counted as secondary cases. Exclusion criteria were single person households, households in which a household contact developed symptoms prior to the primary case and cases for which no household information was known. Sibling pair families were combined. Secondary attack rate (SAR) was calculated as the number of cases divided by the persons at risk.

### Testing strategy

According to national outbreak management protocols for primary schools, classes are put into quarantine for ten days following a single case visiting the classroom from two days before symptom onset [6]. All children from affected classes including teachers are recommended to be tested twice, i.e., once as soon as possible after exposure, and once five days after exposure. If several classes are affected, closure of the entire school may be considered. Whole genome sequencing was performed on random samples from at least one child and one teacher of each affected class. In classes with more than 10 cases we tried to sample additional samples. Antigen tests and samples with a cycle threshold value > 32 were excluded as whole genome sequencing (WGS) was not possible. Samples not investigated at the Maastricht University Medical Centre (MUMC +) were requested at the relevant laboratories.

### Laboratory testing

Laboratory confirmation of SARS-CoV-2 was performed via an RT-PCR assay or antigen test on a nasopharyngeal sample. Samples investigated in the MUMC + were determined using the following method. First, for RNA extraction, 900 µl of clinical sample were mixed with 900 µl of Chemagic Viral Lysis Buffer (Perkin-Elmer) and RNA was extracted from samples using the Chemagic Viral DNA/RNA 300 Kit H96 (Perkin-Elmer) on the Chemagic 360 system (Perkin-Elmer). A multiplex RT-PCR was performed using the N1-gene and E-gene as targets, including the immediate early gene of mouse cytomegalovirus as an internal control. cDNA synthesis and PCR amplification were combined using the TaqPath™ 1-Step RT-qPCR Master Mix, CG (Applied Biosystems, US). Thermal cycling was performed using the Quantstudio 5 Real-Time PCR System (Applied Biosystems, US). Oligonucleotides were synthesised and provided by Biolegio (Netherlands).

### Whole genome sequencing

Samples that were tested positive for SARS-CoV-2 were stored at -80 degrees Celsius until RNA was isolated for sequencing. For RNA extraction, 90 µl of sample were mixed with 90 µl of Chemagic Viral Lysis Buffer (Perkin-Elmer), followed by extraction using the MagNA Pure 96 DNA and Viral NA Small Volume Kit 96 (Roche, Germany) on the MagNA Pure 96 system (Roche, Germany), without the addition of an internal extraction control.

Sequencing was performed using the PCR tiling of SARS-CoV-2 virus with Native Barcoding Expansion 96 (EXP-NBD196) protocol (Version: PTCN_9103_v109_revH_13Jul2020) of Oxford Nanopore technologies, with minor modifications and using the primers previously published by Oude Munnink et al. [7]. Briefly, the only modifications were extending the barcode and adaptor ligation steps up to 60 min and loading 48 samples per flow cell.

Bioinformatic analysis was performed using an in-house developed pipeline MACOVID v2.0 that is based on Artic v1.1.3. In brief, short and obvious chimeric reads are filtered with Cutadapt v2.5. The filtered reads were mapped to the reference genome MN908947.3 with Minimap2 v2.17 and quality checked with “align_trim” function of Artic v1.1.3. Mapped reads were split per primer pool using Samtools v1.9 and a consensus was created per primer pool with Medaka v1.0.3. Variants were called using Medaka v1.0.3 and Longshot v0.4.1. Low coverage regions (< 30x) were masked with “artic_make_depth_mask” function of Artic v1.1.3. A preconsensus was made with “artic_mask” and the final consensus sequence was made with bcftools v1.10.2. Documentation and source code are available from https://github.com/MUMC-MEDMIC/MACOVID under MIT license. The consensus sequences were used to construct a phylogenetic tree with ncov pipeline (https://github.com/nextstrain/ncov) v3 of nextstrain with all B.1.1.7 and Dutch genomes in the Global Initiative on Sharing All Influenza Data (15-apr-2021) as a reference. The WGS used in this paper was considered to be highly accurate as a 100% score was obtained in identifying single nucleotide polymorphisms and indels, Pango lineages and clusters between samples in an external quality assessment of SARS-CoV-2 WGS across 15 European Laboratories[8].

All unsuccessful sequenced samples were analyzed a second time to increase the success rate of the sequencing procedure. For cluster identification, a cut-off value of ≤ 2 single site mutations difference between genomes was applied. Isolates with genomic mutations within this cut-off were deemed be part of the same genotypic cluster, hereafter referred to as the same genotype. All FASTA files had been uploaded to GISAID (see Supplementary file 1 for accession numbers).

## Results

### Primary cases

Three outbreaks occurred in school 1, 2 and 3 starting in March 2021 with affected children from multiple classes and age groups, as well as staff members. Most teachers taught one class but in all schools some teachers taught several classes. None of the teachers had been fully vaccinated against COVID-19. School 1 and 3 had no mechanical ventilation system in place. All schools increased ventilation by opening windows and doors during class. Students from school 2 were able to enter their classrooms directly from outside bypassing the corridors. School 2 and 3 consisted of two buildings, with a separate building for grades 1–3 in school 2 and grades 1–4 in school 3.

The three outbreaks comprised 121 cases, 21 staff and 100 students. Students were 51% male (51/100), with a mean age of 9.4 years, and 28% (28/100) were asymptomatic. Table 1 provides an overview including genotypes determined in each class. Overall AR in children was 12% (n = 33/265), 12% (n = 32/270) and 7% (n = 36/529) in school 1, 2 and 3, respectively, with peak AR of 53% (n = 17/32), 36% (n = 10/28) and 42% (n = 14/33) in individual classes per school. AR among staff was 60% (n = 9/16), 39% (n = 7/18) and 14% (n = 5/35). In classes reporting > 2 cases (n = 11), 46% (5/11) had no teacher testing positive. No clear index case was identified in any of the outbreaks. Nine sibling pairs comprising 7 households were identified among cases of whom only one sibling pair, consisting of a child from class 4B and 6B of school 3, both attended the school during their contagious period.

**Table 1:** Attack rates and SARS-CoV-2 genotypes in schoolchildren and staff, per school and class

	School 1					School 2					School 3				
Class	Children	AR	GT	Positive teacher	GT	Children	AR	GT	Positive teacher	GT	Children	AR	GT	Positive teacher	GT
1A (age 4–5)	–	–	–	–	–	1/32	3%	-	No	–	–	–	–	–	–
1/2A (age 4–6)	0/27	0%	–	No	–	2/22	9%	N/A	Yes	N/A	1/84*	1%	E	No	–
1/2B	2/27	7%	A, A	No		–	–	–	–	–	–	–	–	–	–
1/2C	1/29	4%	A1**	Yes	A	–	–	–	–	–	–	–	–	–	–
2/3A (age 5–7)	–	–	–	–	–	2/26	8%	C	No	–	2/52*	4%	N/A	No	–
3A (age 6–7)	0/26	0%	–	No	–	–	–	–	–	–	0/63*	0%	–	–	–
4A (age 7–8)	1/29	3%	N/A	Yes	A	6/35	17%	N/A	Yes	C	–	-	–	–	–
4B	–	–	–	–	–	–	–	–	–	–	3/30	10%	D, F	No	
5A (age 8–9)	6/31	23%	A1**	No		1/33	3%	C1*	No	–	0/48*	0%	–	No	–
6A (age 9–10)	17/32	53%	A	Yes	A	1/21	5%	C	Yes	C	4/34	12%	G	No	–
6B	–	–	–	–	–	1/19	5%	-	No	–	13/33	39%	D, D	Yes****	D, D
7A (age 10–11)	3/32	9%	A, B	No		10/28	36%	C	Yes	N/A	0/27	0%	–	No	–
7B	–	–	–	–	–	–	–	–	–	–	6/27	22%	D	No	–
7C	–	–	–	–	–	–	–	–	–	–	2/28	7%	N/A	No	–
8A (age 11–12)	2/32	6%	A2***	Yes	A	8/26	35%	C	Yes	C	4/25	16%	D	Yes	D
8B	–	–	–	–	–	–	–	–	–	–	1/26	4%	D	No	–
8C	–	–	–	–	–	–	–	–	–	–	0/24	0%	–	No	–
Total children	32/265	12%				32/270	12%				36/529	7%			
Total staff	9/15	60%				7/18	39%				5/35	14%			

All sequenced samples were variant B.1.1.7. *AR* attack rate, *GT* genotype, *N/A* Not available. *Multiple classes from the same grade grouped together. **This isolate harbored 1 additional mutation compared to the other sequences in this cluster. ***This isolate harbored 2 additional mutations compared to the other sequences in this cluster. ****Four teachers tested positive

**Fig. 1:**
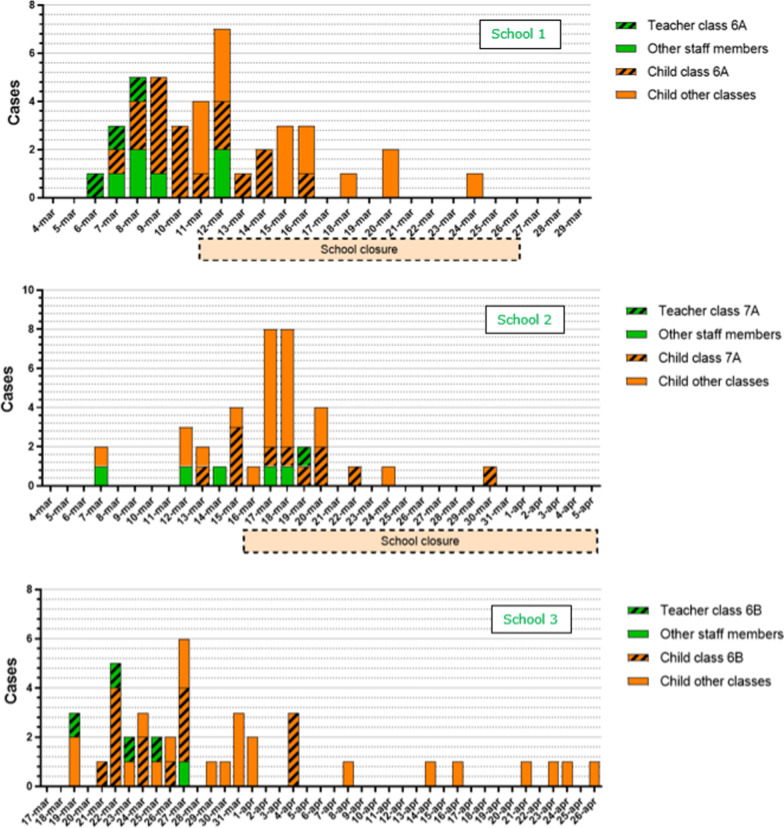
Epidemic curves of each outbreak split per school including duration of school closures. Staff members and children are displayed separately. Cases originating from the class with the highest PAR are accentuated

Figure 1 shows epidemic curves of all three outbreaks including the timing of school closures. Cases from the class with the highest AR are accentuated. An overview of cases, their respective classes and day of symptom onset are given in Additional file 1.

***School outbreak 1*** consisted of 32/265 positive children (AR 12%) and 9/15 staff members (AR 60%). Multiple staff members and children reported symptoms from March 6 onwards. Three teachers and one child from grade 6A were tested March 9 and were notified of their positive result March 10. After being notified of these cases the school closed for two weeks. Following the days after closure, children from multiple classes tested positive. Most cases originated from class 6A with a total AR 52% (n = 17/32) with 3 positive teachers, whereas in class 5A (6/31) and 7A (3/32) multiple cases arose among children without any cases in teachers. No further cases were reported in the two weeks after school reopened. Fifteen samples were sent in for sequencing.

***School outbreak 2*** consisted of 32/270 positive children (12%) and 7/18 staff members (39%). One teacher from class 8A developed symptoms March 7 and tested positive March 8. Because he had not visited the school in the two days preceding symptom development he was subsequently put into isolation without additional measures for class 8A. One child from grade 8 developed symptoms March 7 and tested positive March 13. On March 15 the school was notified of two cases among staff and two cases among children, and the school closed the following three weeks. The days after closure children from multiple affected classes developed symptoms or tested positive. Most cases originated from class 7A with a total AR 36% (n = 10/28) among children and one positive teacher. The first child case in 7A reported symptoms March 13 while the class 7A teacher reported symptoms March 19. Nine samples were sent in for sequencing.

***School outbreak 3*** consisted of 36/529 positive children (7%) and 5/35 staff members (14%). Each class was put into quarantine when new cases were reported, starting March 23 when cases from grade 6A, 6B and 7C were reported. Class 8A, 8B and 7B were put into quarantine shortly after, following notification of cases among children. Multiple cases were reported after these classes were put into quarantine. Class 6B reopened March 29 but was put into quarantine again after a new case was established March 30. In total, 13/33 children (40%) and four teachers of class 6B tested positive. In class 7B 7/27 children tested positive without a case among teachers. A student from class 8A first reported symptoms starting March 25, whereas one teacher reported symptoms March 27. Class 2A, 4B and 7C were put into quarantine after being notified of a positive case on April 9, April 18 and April 28 respectively. No further cases were reported and the outbreak was considered over May 11. Eighteen samples were sent in for sequencing.

**Fig. 2:**
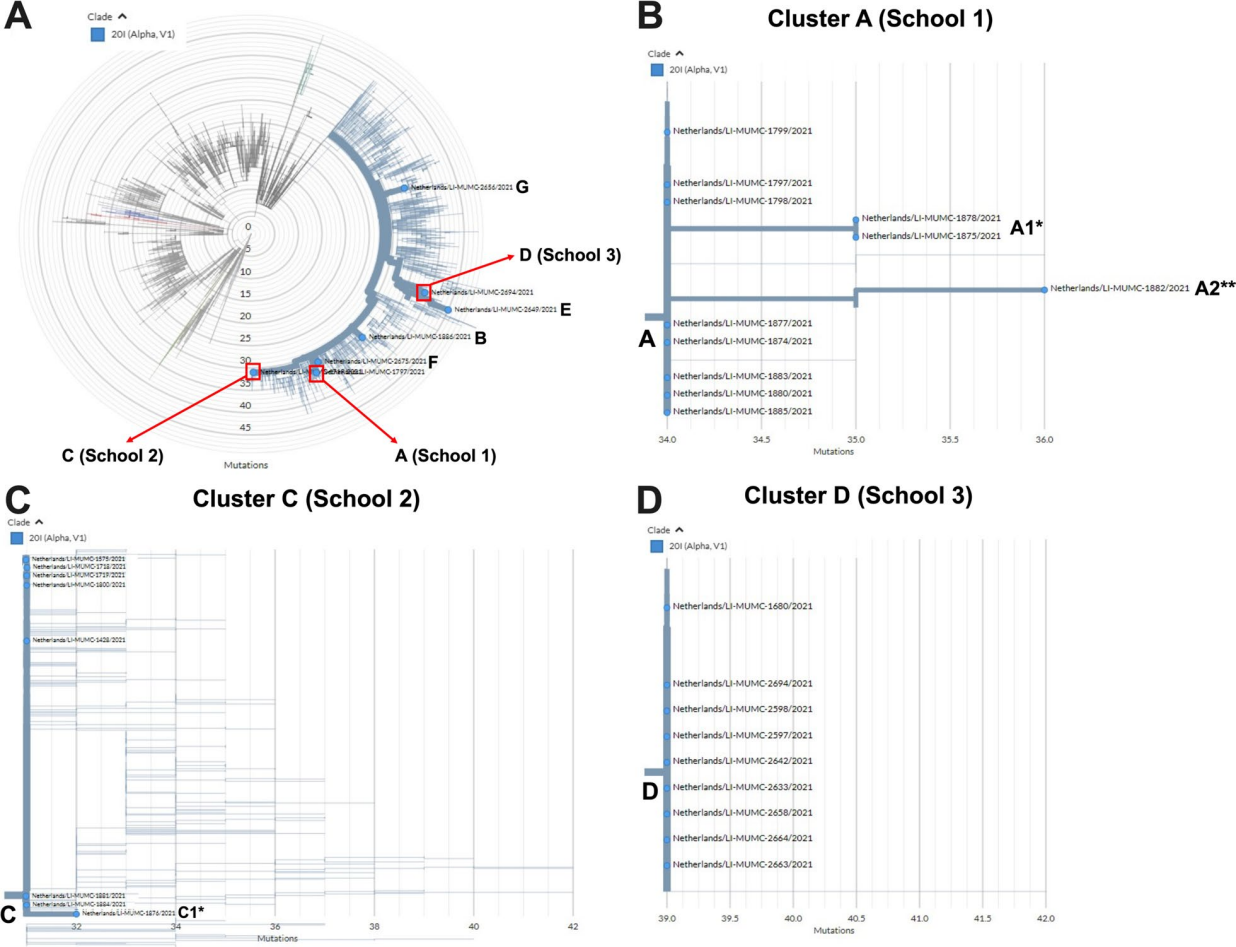
SARS-CoV-2 sequencing results. **A** Overview of the clusters and other sequences identified in this study. One representative sequence is highlighted per sequence cluster. **B** SARS-CoV-2 sequences belonging to sequence cluster A in school 1. **C** SARS-CoV-2 sequences belonging to cluster C in school 2. **D** SARS-CoV-2 sequences belonging to cluster D in school 3. *This isolate harbored 1 additional mutation compared to the other sequences in this cluster. **This isolate harbored 2 additional mutations compared to the other sequences in this cluster. A cut-off value of ≤ 2 mutations difference was applied to consider isolates to be part of the same cluster

***Whole genome sequencing*** was performed successfully on 32/42 (76%) samples, all belonging to alpha variant lineage. School 1, 2 and 3 had 12/15, 8/9 and 12/16 successful sequences respectively. Details on the cases which samples have been (un)successfully sequenced are given in supplementary file 1. The majority of these isolates could be further subdivided in 3 independent sequence clusters (A, C & D), corresponding to the 3 different school outbreaks and one unrelated isolate from a child in school 1 (indicated by a B in Fig. 2(1)) and 3 unrelated isolates from children in school 3 (indicated by E, F & G in Fig. 2). Few other regional genotypes were identified in school 1 and 3, but school 2 harbored many regionally circulating genotypes identified by surveillance. suggesting multiple independent introductions due to spillover from the community or vice versa.

### Secondary cases

Of the 121 cases, 97 households were included for analysis of the SAR. Table 2 provides an overview of the SAR among household contacts. Overall SAR was 40% (99/249, 95% CI 34–36). SAR among symptomatic child household contacts was 39% (85/216, 95% CI 33–46%) and 42% among symptomatic staff household contacts (13/31, 95% CI 25–61%). SAR among asymptomatic children and staff was lower at 9% and 0% respectively.

**Table 2:** Secondary attack rates among household contacts stratified by household type (children’s households versus staff households) and presence of symptoms

Household type (n)	N cases/n household contacts	SAR (95% CI)
Total households (97)	99/249	40% (34–46)
Child households (79)	85/216	39% (33–46)
Symptomatic children (58)	80/164	49% (41–57)
Asymptomatic children (21)	5/52	9% (3–21)
Staff households (18)	14/33	42% (25–61)
Symptomatic staff (16)	13/27	48% (29–68)
Asymptomatic staff (2)	0/4	0% (0–60)

*SAR* secondary attack rate, *CI* confidence interval

### Cases among children increased relative to community incidence

**Fig. 3:**
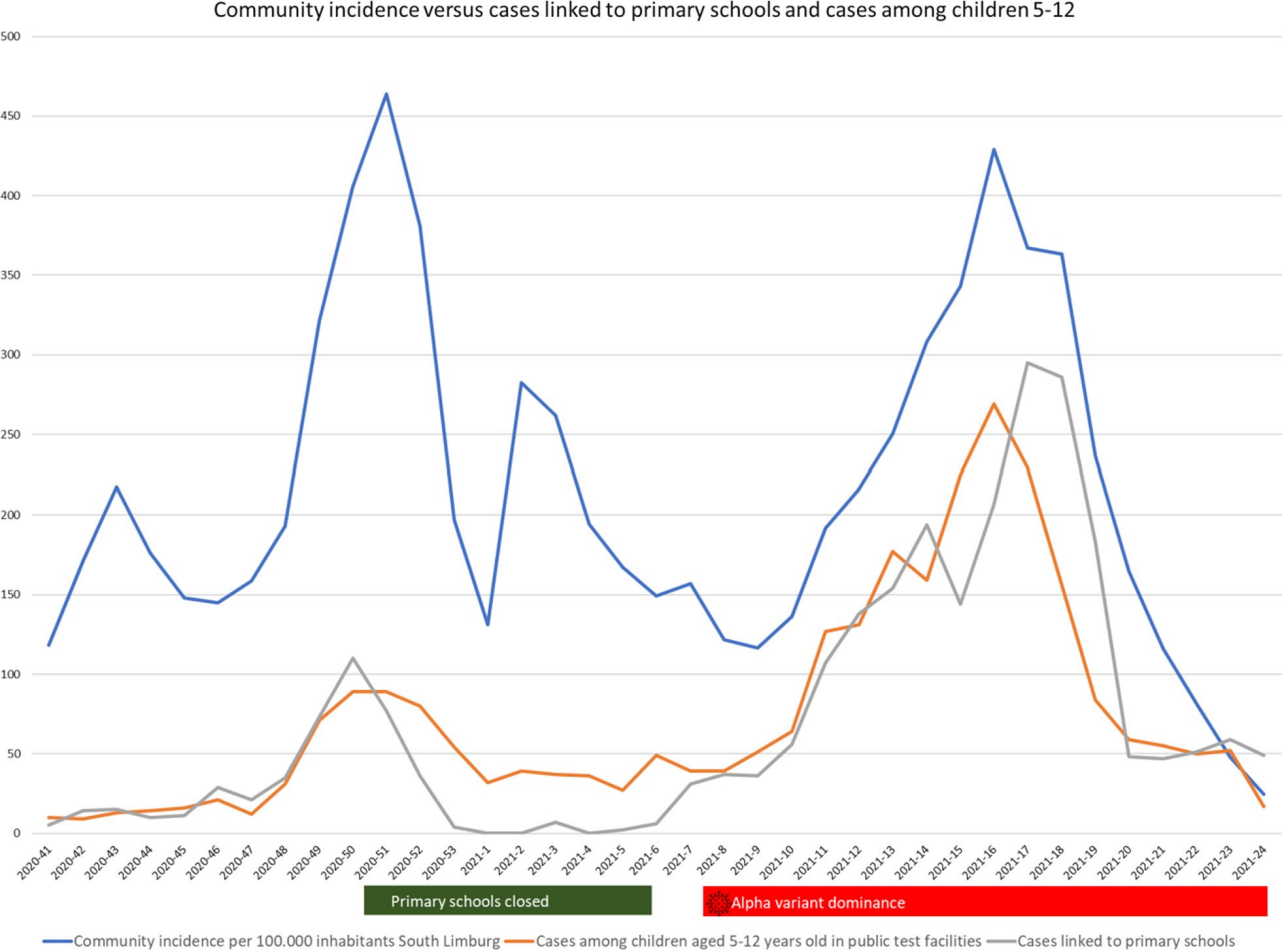
Weekly community incidence per 100.000 inhabitants in South Limburg, Netherlands, week e1 2020 through week 24 2021. Blue: total incidence per 100.000 inhabitants. Orange: number of children aged 5–12 testing positive in public test facilities. Grey: number of cases linked to primary schools. Period of primary school closures and alpha variant dominance (i.e. more than 50% of all cases) indicated by green and red bar, respectively

Figure 3 shows community incidence per 100.000 inhabitants in the region of South Limburg, cases linked to primary schools through source and contact tracing and cases among children 5–12 years old diagnosed in public testing facilities in the study region. From February 22 onwards the alpha variant comprised more than 50% of all cases. Relative to the community incidence, both the number of cases linked to primary schools and cases among children aged 5–12 was higher during the period of alpha variant dominance compared to the earlier surge in December 2021 prior to the closing of schools.

## Discussion

Our analysis of three outbreaks with the alpha variant in primary schools shows high ARs and SARs with evidence of large-scale transmission among children, staff members and their household contacts. The role of child-to-child transmission appeared more prominent than in previous studies featuring wild type original strain outbreaks, suggesting increased infectiousness of the alpha variant in children [2]. High SAR among child household contacts, similar to those among staff household contacts, suggests similar infectivity of children compared to adults. Our findings show that effective infection prevention and control in primary schools is challenging, arguing for intensified measures including universal masking, further improving ventilation and physical distancing.

Earlier studies on outbreaks in schools with the wild type original strain suggest transmission between staff members and staff-to-child plays a dominant role in transmission with low child-to-child transmission [9]. A meta-analysis determined that school staff have an increased chance of being infected compared to the general population [10]. While a higher AR observed among staff members compared to children suggests that staff-to-staff transmission played an important contributory role, we found ample evidence of child-to-child transmission, likely due to increased infectiousness of the alpha variant in children. In 5/11 (46%) classes with > 2 cases no teacher tested positive. Additionally, class 7A of school 2, the most affected class with a AR of 36%, had multiple children testing positive preceding symptom development of their teacher. This is in agreement with a recent study on primary school children that determined many examples of child-to-child transmission [11]. The high SAR among children’s household contacts, similar to the SAR in household contacts of staff members, also points towards increased infectiousness in children. SARs were higher than described in pre-VOC studies but similar to findings from a study on outbreaks in child day care centers and a study in households, both conducted during alpha variant dominance [4, 12, 13]. Our findings are mirrored by an increase in cases among children aged 5–12 and cases linked to primary schools by source and contact tracing during alpha variant dominance suggesting increased transmission at schools.

The difference in SAR among household contacts of symptomatic cases and asymptomatic cases suggests decreased infectiousness in asymptomatic cases relative to symptomatic cases. Our findings are in agreement with an earlier study that found a lower SAR in the school setting for asymptomatic cases [14].

On-site school visits and interviews by the Public Health Service provided insight into factors that may have also contributed to transmission. Among these, lack of mandatory masking, insufficient ventilation and lack of physical distancing appear to have played a prominent role. Masking was only required for staff members outside the classrooms and not for children. A large study from the USA during dominance of the alpha variant found low secondary attack rates in schools with masking mandatory for both students and staff members [15]. Additionally, one study from Georgia USA found decreased COVID-19 incidence when school staff members were mandated to use masks [16]. Additionally, a large outbreak in Israel is linked to incorrect mask use during a heat wave [17]. Universal masking is currently recommended by the CDC [18] and could be considered in the Netherlands to prevent transmission.

Improving ventilation conditions in classrooms resulted in a 37–48% lower COVID-19 incidence in schools in Georgia, USA and a modelling study determined that a combination of natural ventilation, masks and HEPA filtration can decrease the cumulative dose of viruses absorbed by exposed occupants 30 fold [16, 19]. While all schools improved ventilation by frequently opening windows and doors a lack of a mechanical ventilation system in school 1 and 3 could have contributed to transmission. More research is necessary for determining optimal ventilation requirements in classrooms.

Mixing of children of different classes during lunchbreaks or when entering the building may have facilitated transmission among classes in school 3. Lastly, extracurricular activities like sports could have contributed. Sport-associated exposure, especially indoor, has been associated with a high SAR. While indoor sport activities were banned during our study period, alternative transmission routes outside of school, e.g., during leisure time activities including outdoor sports activities, and adherence to guidelines prevention measures were not assessed, giving an incomplete view of transmission routes. The genotype associated with school 2 appeared to be highly prevalent in specimens randomly selected for regional community surveillance so multiple independent community introductions from the community could also partially explain this outbreak. While alternative transmission routes may have contributed its overall effect was probably low as transmission occurred rapidly in all affected schools suggesting transmission primarily occurred at school.

Rapid school closures following first case notification was highly effective curbing further transmission, as did quarantining of individual classes in school 3. School closures should be a last resort and should only be considered when a majority of classes is affected. The high AR in primary school outbreaks and high SAR among household contacts suggest these settings may play an important role in facilitating community transmission. Additionally, VOC B.1.617.2, now commonly referred to as the delta variant and currently the dominant strain in the Netherlands, is estimated to be 55% more transmissible and contains four fold higher viral loads than the alpha variant, lending additional urgency to adequate approaches in school settings [20, 21]. Universal school closures have a negative effect on learning and mental wellbeing of children and should be prevented [22]. The exact role of potential contributors facilitating transmission and effective outbreak control measures should be assessed by further research.

A strength of this study is the detailed investigation of multiple outbreaks with on school visits and whole genome sequencing results towards a detailed background of primary school cases and community incidence. However, our study has several limitations. First, in only a subset of cases was whole genome sequencing performed so it remains unclear if all children have the same sequence type. Because in most affected classes a similar sequence type was determined we don’t think this impacted our main findings. Secondly, multiple independent introductions with a similar sequence type cannot be excluded in the schools but given the speed of the outbreaks we think most transmission occurred at the schools. Thirdly, no data on negative tests is available. Interviews with schools suggest many but not all children or household contacts were tested during the outbreak. This could have led to an underestimation of our AR and SAR and only strengthens the validity of our results. Fourthly, no sequencing has been performed on household contacts so exact transmission routes in households remains unclear. Because household in which an adult had an earlier date of symptom onset were excluded and families were recommended to quarantine following notification of a case in the household we think transmission outside the household did not significantly influence our calculated SAR. Lastly, national guidelines on testing children changed over the course of the pandemic possibly introducing bias in our comparison of cases before and during alpha variant dominance but the overall trend of more cases reported among younger children remains.

## Conclusion

We report three school outbreaks of the alpha variant of SARS-CoV-2 with evidence of large-scale transmission among children, staff members and their household contacts, with high AR’s and SAR’s. Our findings suggest that child-to-child transmission in schools and transmission from children to household contacts may play a larger role than hitherto described in studies predating the appearance of VOC’s. New and more transmissible variants may further increase transmission at primary schools.

## Supplementary Information

**Additional file 1. **An overview of cases, their respective classes, day of symptom onset and genotypes.

## Data Availability

Nucleic acid sequence data has been shared with the Global Initiative on Sharing All Influenza Data database. In the supplementary material an overview of all samples is provided with corresponding accession numbers.

## Abbreviations

VOC: Variant of concern
SAR: Secondary attack rate
AR: Attack rate
RT-PCR: Real-time polymerase chain reaction
MUMC +: Maastricht University Medical Centre
WGS: Whole genome sequencing
CI: Confidence interval
